# The Procedureless Elipse Gastric Balloon Program: Multicenter Experience in 1770 Consecutive Patients

**DOI:** 10.1007/s11695-020-04539-8

**Published:** 2020-04-11

**Authors:** R. Ienca, Mohammed Al Jarallah, Adelardo Caballero, Cristiano Giardiello, Michele Rosa, Sébastien Kolmer, Hugues Sebbag, Julie Hansoulle, Giovanni Quartararo, Sophie Al Samman Zouaghi, Girish Juneja, Sébastien Murcia, Roman Turro, Alberto Pagan, Faruq Badiuddin, Jérôme Dargent, Pierre Urbain, Stefan Paveliu, Rita Schiano di Cola, Corrado Selvaggio, Mohammed Al Kuwari

**Affiliations:** 1 Weight Management Center, Nuova Villa Claudia Clinic, Rome, Italy; 2 General Surgery Department, Jarallah German Clinic, Kuwait City, Kuwait; 3 Bariatric Center, Instituto De Obesidad, Madrid, Spain; 4 Emergency and Metabolic Surgery Department, Pineta Grande Hospital, Caserta, Italy; 5 Nutritional Center, Micros Clinic, Modica, Italy; 6 Digestive Surgery Department, Le Réseau Pondera, Mulhouse, France; 7 Digestive Surgery Department, Polyclinique Du Parc Rambot, Aix-en-Provence, France; 8 Nutrional Center, Claris Clinic, Brussels, Belgium; 9 General and Bariatric Surgery Unit, Villa Donatello, Florence, Italy; 10 Digestive and Bariatric Surgery Department, Infirmerie Protestante, Caluire, France; 11 Bariatric and Weight Loss Center, Cocoona Center, Dubai, UAE; 12 Bariatric Surgery Department, Nouvelle Clinique Bordeaux Tondu, Floirac, France; 13 Digestive Endoscopy Department, Centro Medico Teknon, Barcelona, Spain; 14 Nutritional Center, Centro Integral Nutricion Baleares-Cinib, Palma de Mallorca, Spain; 15 General and Obesity Surgery Department, BR Medical Suites, Dubai, UAE; 16 Bariatric Surgery Department, Polyclinique Lyon Nord, Rillieux-la-Pape, France; 17 General Surgery Department, Polyclinique Saint Privat, Boujan-sur-Libron, France; 18 General and Obesity Surgery Department, Centre Medical Matisse, Nice, France; 19 Bariatric Surgery Department, The Masters Medical Clinic, Doha, Qatar

**Keywords:** Multicenter study, Weight loss, Intragastric balloon, Elipse balloon, Obesity, Overweight, Swallowable balloon, Non-endoscopic balloon, Procedureless, Allurion balloon

## Abstract

**Purpose:**

The Elipse balloon is a novel, non-endoscopic option for weight loss. It is swallowed and filled with fluid. After 4 months, the balloon self-empties and is excreted naturally. Aim of the study was to evaluate safety and efficacy of Elipse balloon in a large, multicenter, population.

**Materials and Methods:**

Data from 1770 consecutive Elipse balloon patients was analyzed. Data included weight loss, metabolic parameters, ease of placement, device performance, and complications.

**Results:**

Baseline patient characteristics were mean age 38.8 ± 12, mean weight 94.6 ± 18.9 kg, and mean BMI 34.4 ± 5.3 kg/m^2^. Triglycerides were 145.1 ± 62.8 mg/dL, LDL cholesterol was 133.1 ± 48.1 mg/dL, and HbA1c was 5.1 ± 1.1%. Four-month results were WL 13.5 ± 5.8 kg, %EWL 67.0 ± 64.1, BMI reduction 4.9 ± 2.0, and %TBWL 14.2 ± 5.0. All metabolic parameters improved. 99.9% of patients were able to swallow the device with 35.9% requiring stylet assistance. Eleven (0.6%) empty balloons were vomited after residence. Fifty-two (2.9%) patients had intolerance requiring balloon removal. Eleven (0.6%) balloons deflated early. There were three small bowel obstructions requiring laparoscopic surgery. All three occurred in 2016 from an earlier design of the balloon. Four (0.02%) spontaneous hyperinflations occurred. There was one (0.06%) case each of esophagitis, pancreatitis, gastric dilation, gastric outlet obstruction, delayed intestinal balloon transit, and gastric perforation (repaired laparoscopically).

**Conclusion:**

The Elipse™ Balloon demonstrated an excellent safety profile. The balloon also exhibited remarkable efficacy with 14.2% TBWL and improvement across all metabolic parameters.

## Introduction

The obesity epidemic is now a worldwide phenomenon. Diet and exercise have been ineffective in controlling this epidemic. Bariatric surgery, although effective, has significant associated risks. Minimally invasive techniques, including endoscopically placed gastric balloons, have been introduced to provide a safer alternative for achieving weight loss. The new swallowable gastric balloon, Elipse® (Allurion Technologies, Natick, MA, USA), represents an innovative option for weight loss that does not require endoscopy or anesthesia. Several studies have shown it to be a simple, safe, and effective method for weight loss [1–5]. Although the residence time of Elipse in the stomach is less than other conventional intragastric balloons (IGBs) that need endoscopy, the results appear to be comparable [1–3]. In addition, the non-invasive nature of the Elipse method enables new treatment paradigms for multiple categories of patients; overweight patients, who otherwise would not elect a more invasive treatment, may choose Elipse, and patients with higher BMI, who fear anesthesia, may pursue multiple Elipse balloon treatments in series. The efficacy of consecutive IGB treatments in morbidly obese patients has already been described in several studies [6–9]. Recently, endoscopic treatment with IGBs has also emerged as a therapeutic option for adolescents [10, 11] and the Elipse device could represent a more approachable tool for weight loss in this difficult-to-manage category of patients. Moreover, the easier administration of the Elipse balloon and the absence of endoscopy for placement or removal allow the extension of its use not only to the surgeon and the endoscopist but also to other obesity specialists. In fact, in this study, over 1700 patients were enrolled at nineteen obesity centers of excellence led not only by surgeons and endoscopists but also by obesity clinicians who are specialists in longitudinal weight loss management.

### Aim

The primary objectives of this study were to confirm the Elipse gastric balloon system’s safety and evaluate the mean weight loss of the Elipse balloon for the treatment of overweight and obese individuals in a large, multicenter, diverse international population.

### Patients and Methods

#### The Elipse Gastric Balloon System

**Fig. 1:**
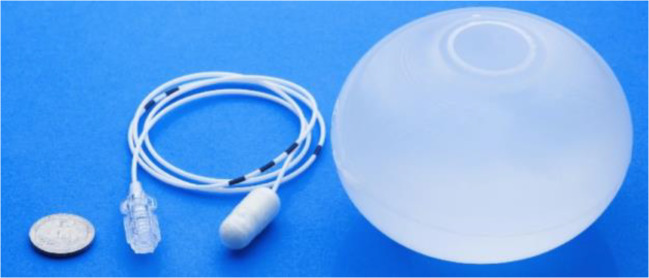
The Elipse® balloon

**Fig. 2:**
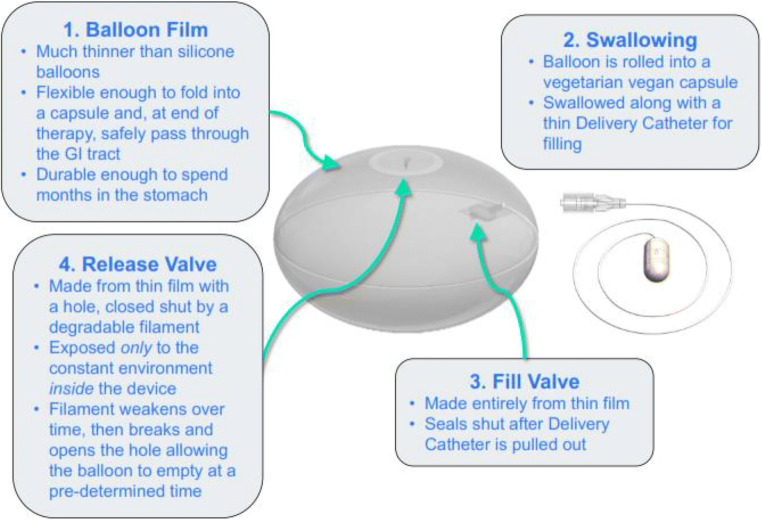
Elipse: Key innovations

**Fig. 3:**
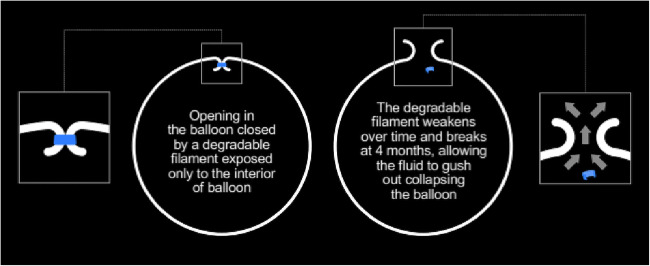
Mechanism of valve opening

The Elipse balloon (Allurion Technologies, Natick, MA, USA) is compressed into a swallowable vegan capsule connected to a thin catheter (Fig. 1), through which the balloon is filled with 550 mL of liquid after it reaches the stomach. Placement is performed in a 20-min outpatient visit without endoscopy or sedation. After filling and once the correct position of the balloon is confirmed via an abdominal X-ray, the thin catheter is removed (Fig. 2). At approximately 4 months, a valve in the Elipse balloon spontaneously opens (Fig. 3), the balloon empties, and it is then excreted through the gastrointestinal tract. A thin guidewire acting as a stylet (to slightly stiffen the catheter) may be used in case of difficulty swallowing.

**Fig. 4:**
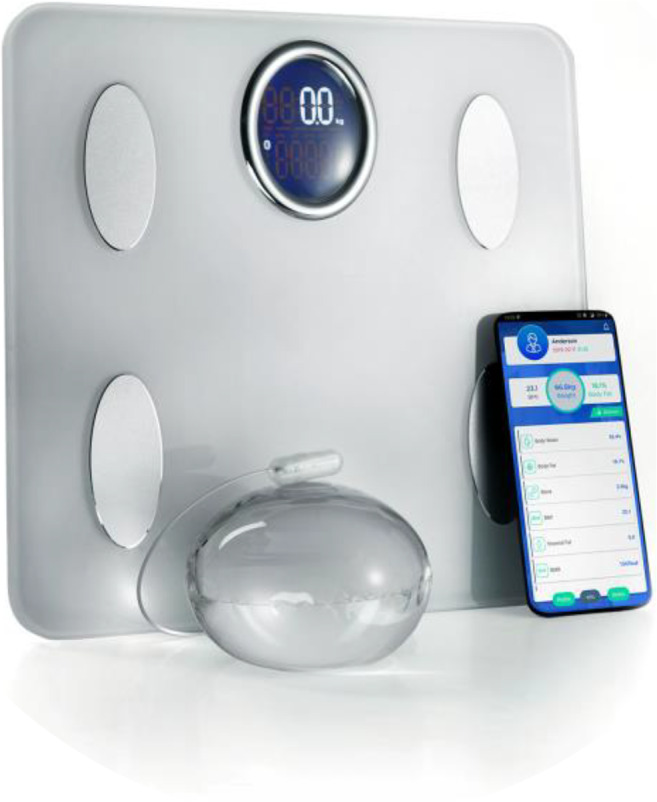
The Elipse system: Elipse balloon, Bluetooth® body composition scale, and smartphone app for virtual follow-up

The Elipse balloon is accompanied by a wireless, Bluetooth®-enabled body composition scale and a smartphone application that enable weight loss tracking and communication between the patient and his/her care team (Fig. 4).

### Study Design and Patients

#### Hypothesis/Determination of the Sample Size

Sample size justification: A sample size of 1500 provides 80.9% power to exclude the probability of an event rate of 0.5% if the true event rate is 0.1% or less, assuming a 1-sided alpha of 0.025, with H_0_: Event rate ≥ 0.5%; H_a_: Event rate < 0.5%. In addition, 1500 subjects provide adequate sample size for key demographic subsets.

### Efficacy Endpoints

- Weight loss (kg): Weight loss is calculated as Month 4 weight (kg) − Baseline weight (kg).
- Percent total body weight loss (%TBWL): %TBWL is calculated as [(Month 4 weight (kg) − Baseline weight (kg))/(Baseline weight (kg))] × 100%.
- BMI loss (kg/m^2^): BMI loss is calculated as Month 4 BMI (kg/m^2^) − Baseline BMI (kg/m^2^).
- Percent excess weight loss (%EBWL): %EBWL is calculated using a reference normal BMI of 25 kg/m^2^, as {[(Baseline BMI-25) − (Month 4 BMI-25)]/(Baseline BMI-25)} × 100%.
- Change in laboratory values: For each laboratory value of triglycerides (mg/dL), LDL (mg/dL), and HbA1c (%), the difference between Month 4 and Baseline was calculated.

For each endpoint, subset analysis was performed by gender and BMI (kg/m^2^) (< 30, 30 to 40, and > 40).

Schedule of assessments

**Table:** 

Procedures	Baseline (screening and enrollment)	Elipse treatment	1-month follow-up visit	2-month follow-up visit	3-month follow-up visit	4-month follow-up visit	Device elimination follow-up visit/registry exit
Medical and obesity history	X						
Focused physical exam	X						
Waist circumference	X	X	X	X	X	X	
Height	X						
Weight—office visit	X	X	X	X	X	X	
Nutrition counseling	X	X	X	X	X	X	
Elipse treatment		X					
Fluoroscopy or X-rays		X^1,2^					
Abdominal exam	X						
Blood tests (triglycerides, LDL, and HbA1c)^3^	X					X	X
Safety evaluation		X	X	X	X	X	X

^1^Once the device is swallowed, fluoroscopy or abdominal X-ray is used to confirm that the capsule is within the stomach prior to filling the balloon

^2^Once filling is complete, either fluoroscopy or abdominal X-ray is performed in order to confirm the balloon position within the stomach

^3^To be collected in a subset of patients

### Safety Endpoint

The safety endpoint was measured via the collection of adverse event and complication data associated with the use of the Elipse system. Data were analyzed to evaluate continued acceptability of identified risks and detection of emerging risks on the basis of the data.

This was a multicenter, prospective, non-randomized, open-label, registry study conducted in overweight and obese patients from January 2016 until June 2019. Nineteen international obesity centers were involved in the study. Data collection started on January 2016. Each of the nineteen obesity center, before starting to use Elipse, received a custom-made database aimed to collect the most pertinent information related to the Elipse treatment program. The data was collected prospectively by pre-identified personnel at each study site with experience in data collection. Once submitted to the author coordinator (author 1), she merged all the data to perform data analysis. Inclusion criteria were age between 18 and 65 years and body mass index (BMI) greater than 27 kg/m^2^ with previous failed dietary treatments. Key contraindications for the study included pregnant women, patients with a history of three or more caesarean sections, patients with swallowing problems, patients with previous intestinal obstruction, patients with voluminous hiatal hernia (larger than 4–5 cm), and those with GI cancer and GI bleeding, severe coagulopathy, or severe psychological or eating disorders. Conditions that predispose to bowel obstruction (history of perforated appendicitis; history of abdominal or pelvic surgery excluding any single one, but not more than one, of the following surgeries that was performed at least 12 months prior to Elipse treatment: diagnostic laparoscopy, laparoscopic appendectomy, open appendectomy with a right lower quadrant incision, laparoscopic cholecystectomy; inflammatory bowel disease: Crohn’s disease and ulcerative colitis; severe GI motility disorder such as severe gastroparesis); and conditions that predispose to gastric perforation (history of previous gastric or esophageal surgery; history of previous laparoscopic band ligation; history of anti-reflux surgery).

To prevent an increase in gastroesophageal reflux discomfort, patients start the prophylactic therapy with PPI 2 weeks before the placement and continue this therapy during the 4 months of balloon residence in the stomach. Based on the intensity of reflux symptoms reported by any patient during the screening visit, each physician may consider the option to perform an endoscopy before the placement to evaluate the exact condition of the esophagus and stomach. If there were any symptomatology that suggests a gastroesophageal problem such as abdominal pain, persistent or severe reflux, and abdominal tenderness, an endoscopy or imaging to evaluate is always performed.

### Intervention

The Elipse balloon was placed in 1770 overweight and obese patients (F 1264/M 506) at 19 international obesity centers of excellence (Table 1). During the Elipse program, the patients were closely followed by a dedicated multi-disciplinary team. The program commenced 2 weeks prior to balloon placement and continued until balloon passage at approximately 4 months. Prior to placement, a detailed medical obesity history, nutritional behavior history, and anthropometric evaluation (height, weight, BMI, circumference of waist) were performed. Laboratory values were collected in a subset of sites. These sites were chosen by their strong interest in metabolic disorders associated with obesity. Four hours prior to the deployment of the balloon, the patients received a single dose (125 mg PO) of the anti-emetic aprepitant (Emend®). Immediately following balloon placement, patients received ondansetron 4 mg PO every 8 h for 3 days. Two more doses of aprepitant (80 mg PO) were prescribed before discharge along with an anti-spasmodic as needed. The patients were treated daily with a proton pump inhibitor (lansoprazole 30 mg/day PO or equivalent PPI) for the entire treatment period, starting 2 weeks before balloon placement to heal any asymptomatic superficial inflammation if present augmenting the safety of the device. The patients were advised to avoid NSAIDs and other gastric irritants during the study. The patients fasted for at least 8 h prior to the placement procedure. Only fluid hydration was permitted for the first 24 h and a gradual progression towards a semi-solid diet, and subsequently solid diet, was carried out by the patients over 1 to 2 weeks. The diets were administered by a nutritionist or dietitian who supported the patients for the entire treatment period. All the patients received a wireless, Bluetooth®-enabled body composition scale and a smartphone app (Fig. 1) that enabled weight loss tracking and communication between the patient and his/her care team. In-person monthly visits were conducted until the end of the program.

**Table 1:** Centers involved in the study

Institute	City
Nuova Villa Claudia	Roma (Italy)
Instituto De Obesidad	Madrid (Spain)
Pineta Grande Hospital	Caserta (Italy)
Micros Clinic	Modica (Italy)
German Clinic	Kuwait (Kuwait)
Le Réseau Pondera	Mulhouse (France)
Polyclinique Du Parc Rambot	Aix-an-Provence (France)
Claris Clinic	Brussels (Belgium)
Villa Donatello	Florence (Italy)
Infirmerie Protestante	Caluire (France)
Cocoona Center	Dubai (UAE)
Centro Medico Teknon	Barcelona (Spain)
Centro Integral Nutricion Baleares-Cinib	Palma de Mallorca (Spain)
Polyclinique Lyon Nord	Rilleuux-la-Pape (France)
Dubai Healthcare City	Dubai (UAE)
Nouvelle Clinique Bordeaux Tondu	Floirac (France)
Polyclinique Saint Privet	Boujan-sur-Libron (France)
Centre Medical Matisse	Nice (France)
The Masters Medical Clinic	Doha (Qatar)

## Results

### Patients

A total of 1770 patients underwent Elipse treatment and received the medication doses as per protocol. Sixty-three patients (3.6%) did not complete the program and had the balloon removed before 4 months due to intolerance or other adverse events.

### Anthropometric and Metabolic Parameters

At baseline, patients showed the following characteristics: mean age 38.8 ± 12, mean weight 94.6 ± 18.9 kg, and mean BMI 34.4 ± 5.3 kg/m^2^. Triglycerides (*n* = 407) were 145.1 ± 62.8 mg/dL and LDL cholesterol (*n* = 407) was 133.1 ± 48.1 mg/dL, while HbA1c (*n* = 391) was 5.1 ± 1.1% (Table 2).

**Table 2:** Patient demographics before Elipse treatment

Sex (F/M)	1264/506
Age (years)	38.8 ± 12
Weight (kg)	94.6 ± 18.9
BMI (kg/m^2^)	34.4 ± 5.3
Triglycerides (mg/dL)	145.1 ± 62.8
LDL cholesterol (mg/dL)	133.1 ± 48.1
HbA1c (%)	5.1 ± 1.1%

### Outcome

After 4 months, overall mean weight loss (WL) was 13.5 ± 5.8 kg, mean percent excess weight loss (EWL%) was 67.0 ± 64.1, and a mean BMI reduction (BMIL) was 4.9 ± 2.0 points. Percentage total body weight loss (TBWL%) was 14.2 ± 5.0 (Table 3). Elipse therapy led to improvements in all the metabolic parameters investigated (Table 4).

**Table 3:** Weight loss results after Elipse treatment

	Mean (SD).
Wl (kg)	13.5 ± 5.8, *p* < 0.0001 (from baseline)
%TBWL	14.2 ± 5.0, *p* < 0.0001 (from baseline)
%EWL	67.0 ± 64.1, *p* < 0.0001 (from baseline)
BMIL (kg/m^2^)	4.9 ± 2.0, *p* < 0.0001 (from baseline)

**Table 4:** Metabolic data results

	Baseline 4	Month results
Triglycerides (mg/dL)	145.1 ± 62.8	99.4 ± 21.8, *p* < 0.0001
LDL (mg/dL)	133.1 ± 48.1	106.9 ± 27.9, *p* < 0.0001
HbA1c (%)	5.1 ± 1.1	4.8 ± 0.8, *p* < 0.0001

### Balloon Performance

At the time of placement, 99.9% of patients were able to swallow the device with 35.9% requiring stylet assistance (Table 5). Eleven (0.6%) empty balloons were vomited at the end of residence time. This was an uncommon method of device passage and was not associated with any adverse events (Table 5).

**Table 5:** Placement and balloon passage

Placement	
Swallowed	1133 (64.0%)
Swallowed with stylet assistance	636 (35.9%)
Placement failed	1 (0.06%)
Balloon passage	
Stool	1692 (95.6%)
Vomited balloon	11 (0.6%)
Endoscopic removals (all causes)	63 (3.6%)
Surgical removals	4 (0.02%)

### Safety

Fifty-two (2.9%) patients had intolerance requiring endoscopic balloon removal. Eleven (0.6%) balloons deflated early and passed uneventfully. Four (0.2%) balloons were endoscopically removed after discovering these patients had prior contraindicated surgery. There were three (0.17%) small bowel obstructions that required laparoscopic surgical removal of the balloon. However, all three occurred early in the study (2016) from an earlier design generation of the balloon. Four (0.02%) events of spontaneous hyperinflation of the balloon occurred. One (0.06%) patient developed esophagitis and one patient pancreatitis (0.06%) both requiring endoscopic balloon removal. One (0.06%) patient had a gastric perforation requiring laparoscopic surgical repair and removal of Elipse. One (0.06%) gastric dilation occurred 15 days after Elipse placement. This resolved by switching the patient from solid to liquid diet for 48 h. Additionally, there were 1 (0.06%) case each of gastric outlet obstruction, requiring endoscopic removal, and delayed intestinal balloon transit (Table 6). There were no thromboembolic complications and deaths in the study.

**Table 6:** Adverse events and complications

Intolerance requiring endoscopic removal	52 (2.9%)
Early deflation (< 3 months)	11 (0.6%)
Spontaneous hyperinflation	4 (0.2%)
Small bowel obstruction	3 (0.17%)
Gastric dilation	1 (0.06%)
Esophagitis	1 (0.06%)
Pancreatitis	1 (0.06%)
Gastric perforation	1 (0.06%)
Delayed intestinal transit	1 (0.06%)
Gastric outlet obstruction	1 (0.06%)

## Discussion

Intragastric balloons have offered a less invasive alternative to surgery for overweight and obese individuals. Although more effective than drugs, diet, and exercise, balloon uptake has been limited due to the need for endoscopy for placement and removal [12]. In morbidly obese patients, it is recommended as a less invasive treatment than bariatric surgery [13, 14] and to reduce comorbidities and surgical risk [15, 16]. Although commercially available intragastric balloons are all somewhat different, in general they have been shown to have comparable weight loss results [1–3]. Several studies have now demonstrated that the data on both the efficacy and safety of Elipse balloon compares very favorably with other, longer duration, balloons [1–3, 17, 18]. Orbera balloon, the most widely used endoscopically placed intragastric balloon, remains the closest in size, shape, and function to the Elipse balloon. The largest analysis of Orbera [19] shows an early Orbera balloon removal rate of 7.5%.

This current registry study is the largest prospective study of the Elipse balloon since its commercialization. In fact, this is one of the largest intragastric balloon studies ever performed. The centers included in the study were high volume obesity treatment centers in several different countries throughout Europe and the Middle East. This enabled a geographically and demographically diverse study population. The study demonstrates a mean TBWL of 14.2% and BMI change of 4.9 points in just 4 months of balloon exposure. These results align with current literature demonstrating that 80–90% of weight loss from a 6-month balloon occurs in the first 3 to 4 months of balloon residence after which the weight loss plateaus [20, 21]. Interestingly, in previously reported data, patients treated with the Elipse system (including the scale and smartphone app) sustained 72% of their weight loss 12 months after balloon excretion [5]. This suggests that their weight maintenance may be related to durable physiological changes that may remain after balloon excretion, maintenance of lifestyle changes that may be promoted by ongoing use of the scale and app, or a combination of both.

The results included Elipse’s impact on weight and metabolic parameters along with ease of placement, device performance, and complications. Overall efficacy outcomes are in line or better than the results reported in earlier, smaller Elipse balloon studies (Table 7). Moreover, this study utilized the entire Elipse system, including the balloon, scale, and smartphone app. The addition of these digital tools may work synergistically with the balloon to enhance weight loss during the 4-month balloon period and also assist in weight loss maintenance after balloon excretion. In fact, excluding sixty-three patients (3.6%) that did not complete the program due to intolerance or other adverse events, all other patients completed the follow-up. This high rate of follow-up was achieved in part due to the close loop communication between the patient and the care team (supported with the use of wireless scale and smartphone app) enhancing the quality of follow-up.

**Table 7:** Average weight loss outcomes on Elipse treatment in published studies

	Sample size (*n*)	Weight loss (kg)	BMI loss (kg/m^2^)	TBWL%	EWL%
Current study (2019)	1770	13.5 ± 5.8	4.9 ± 2.0	14.2 ± 5.0	67.0 ± 64.1
Jamal et al. [5]* (2019)	106	N/A**	3.7	10.9	N/A**
Alsabah et al. [4] (2018)	135	13.1 ± 6.1	4.9 ± 2.2	15.1 ± 9.5	N/A**
Raftopoulus et al. [3] (2017)	12	15.4	5.4	14.6	50.2
Machytka et al. [1] (2016)	34	N/A**	3.9 ± 3.1	10 ± 6.6	N/A**
Genco et al. [2] (2017)	38	12.7	4.2	11.6	26

*Values reported are at 3rd and 6th month, because there are no data at 4th month

**Data were reported as initial and final and not in reduction

Subgroup efficacy analysis demonstrates that the %TBWL was similar in the overweight (BMI < 30 kg/m^2^), obese (BMI 30–40 kg/m^2^), and super-obese (BMI > 40 kg/m^2^) populations: 13.3%, 14.4%, and 14.7% respectively (Table 8). Results also were similar in males and females (Table 9).

**Table 8:** Efficacy subgroup analysis

BMI*	% TBWL mean (SD)
< 30 (*n* = 302)	13.3 ± 4.7
30–40 (*n* = 1230)	14.4 ± 4.9
> 40 (*n* = 196)	14.7 ± 4.2

*BMI data not available for 42 patients

**Table 9:** %TBWL on males and females

Sex	% TBWL mean (SD)
Male	13.8 ± 5.2
Female	14.4 ± 5.0

The Elipse balloon was easily swallowed with only 1 patient unable to swallow the device. 35.9% of patients required stylet assistance to aid swallowing. The ease of the placement procedure and the elimination of endoscopy are two key strengths for the Elipse balloon. These aspects were favorably perceived by both the physicians and the patients. The Elipse balloon was very well tolerated with early accommodative symptoms being controlled with a combination of anti-emetic therapy comprising ondansetron and aprepitant. The early removal rate due to intolerance related to Elipse balloon was low at 2.9%. In this study, 95.6% of the balloons transited safely through the gastrointestinal tract and passed in the stool. Eleven empty balloons (0.6%) were vomited at the end of their residence time without any associated adverse events. Serious adverse events were rare and included 3 small bowel obstructions that were managed laparoscopically. However, these occurred with an earlier generation of the Elipse balloon. Following design changes to mitigate this failure mode, no further small bowel obstructions were reported in the 635 patients treated from year 2018 onwards with the new generation of the device. Spontaneous hyperinflation is a known occurrence with all liquid-filled balloons. In this study, four patients (0.2%) presented with mild to moderate intolerance symptoms and were found to have spontaneous hyperinflation on imaging. These balloons were endoscopically removed without complications. An investigation identified the root cause for the hyperinflation resulting in a manufacturing change to the filling fluid mitigating any further incidents in this study.

A subset of centers that had an interest in metabolic disorders associated with obesity also collected metabolic data that is summarized in Table 4. Improvement was observed in all three metabolic parameters measured: LDL, triglycerides, and HbA1c. Previous studies have demonstrated a significant decrease in obesity comorbidities following the extent of weight loss observed in this study [22, 23].

## Conclusion

This prospective, multicenter registry study across 19 international centers in 1770 patients demonstrates the safety and efficacy of the Elipse gastric balloon system, including the balloon, body composition scale, and smartphone app. The 14.2% TBWL compares well with the weight loss achieved by other longer-duration, endoscopic gastric balloons [24]. The ease of use, low rate of serious adverse events, and potentially lower cost of the Elipse system enable much wider application of gastric balloon technology across the overweight and obese population. Furthermore, elimination of endoscopy and sedation for placement and removal may expand use to a wider group of physicians managing overweight and obese individuals.
