# Correction to: The Procedureless Elipse Gastric Balloon Program: Multicenter Experience in 1770 Consecutive Patients

**DOI:** 10.1007/s11695-020-04941-2

**Published:** 2020-08-31

**Authors:** R. Ienca, Mohammed Al Jarallah, Adelardo Caballero, Cristiano Giardiello, Michele Rosa, Sébastien Kolmer, Hugues Sebbag, Julie Hansoulle, Giovanni Quartararo, Sophie Al Samman Zouaghi, Girish Juneja, Sébastien Murcia, Roman Turro, Alberto Pagan, Faruq Badiuddin, Jérôme Dargent, Pierre Urbain, Stefan Paveliu, Rita Schiano di Cola, Corrado Selvaggio, Mohammed Al Kuwari

**Affiliations:** 1 Weight Management Center, Nuova Villa Claudia Clinic, Rome, Italy; 2 General Surgery Department, Jarallah German Clinic, Kuwait City, Kuwait; 3 Bariatric Center, Instituto De Obesidad, Madrid, Spain; 4 Emergency and Metabolic Surgery Department, Pineta Grande Hospital, Caserta, Italy; 5 Nutritional Center, Micros Clinic, Modica, Italy; 6 Digestive Surgery Department, Le Réseau Pondera, Mulhouse, France; 7 Digestive Surgery Department, Polyclinique Du Parc Rambot, Aix-en-Provence, France; 8 Nutrional Center, Claris Clinic, Brussels, Belgium; 9 General and Bariatric Surgery Unit, Villa Donatello, Florence, Italy; 10 Digestive and Bariatric Surgery Department, Infirmerie Protestante, Caluire, France; 11 Bariatric and Weight Loss Center, Cocoona Center, Dubai, UAE; 12 Bariatric Surgery Department, Nouvelle Clinique Bordeaux Tondu, Floirac, France; 13 Digestive Endoscopy Department, Centro Medico Teknon, Barcelona, Spain; 14 Nutritional Center, Centro Integral Nutricion Baleares-Cinib, Palma de Mallorca, Spain; 15 General and Obesity Surgery Department, BR Medical Suites, Dubai, UAE; 16 Bariatric Surgery Department, Polyclinique Lyon Nord, Rillieux-la-Pape, France; 17 General Surgery Department, Polyclinique Saint Privat, Boujan-sur-Libron, France; 18 General and Obesity Surgery Department, Centre Medical Matisse, Nice, France; 19 Bariatric Surgery Department, The Masters Medical Clinic, Doha, Qatar

**Correction to: Obesity Surgery (2020) 30:3354–3362.**

10.1007/s11695-020-04539-8

This article was originally published electronically on the publisher’s internet portal on May 5, 2020 without open access. With the author(s)’ decision to opt for Open Choice the copyright of the article changed on August 6, 2020 to © The Author(s) 2020 and the article is forthwith distributed under a Creative Commons Attribution.

